# A cryptic phosphate-binding pocket on the SPFH domain of human stomatin that regulates a novel fibril-like self-assembly

**DOI:** 10.1016/j.crstbi.2022.05.002

**Published:** 2022-05-18

**Authors:** Koki Kataoka, Shota Suzuki, Takeshi Tenno, Natsuko Goda, Emi Hibino, Atsunori Oshima, Hidekazu Hiroaki

**Affiliations:** a Laboratory of Structural Molecular Pharmacology, Graduate School of Pharmaceutical Sciences, Nagoya University, Furocho, Chikusa-ku, Nagoya, Aichi, 464-8601, Japan; b BeCellBar LLC, Business Incubation Building, Nagoya University, Furocho, Chikusa-ku, Nagoya, Aichi, 464-8601, Japan; c Cellular and Structural Physiology Institute (CeSPI), Nagoya University, Furocho, Chikusa-ku, Nagoya, Aichi, 464-8601, Japan; d Institute for Glyco-core Research (iGCORE), Nagoya University, Furo-cho, Chikusa-ku, Nagoya, 464-8601, Japan

**Keywords:** CD (circular dichroism), NMR (nuclear magnetic resonance), CSPs (chemical shift perturbations), CSTC (chemical shifts temperature coefficients), NOE (nuclear Overhauser effect), STOM (stomatin), PDB (Protein Data Bank), SPFH (stomatin/prohibitin/flotillin/HflK), TEM (transmission electron microscopy), r. m. s. d. (root mean square difference)

## Abstract

Human stomatin (hSTOM) is a component of the membrane skeleton of erythrocytes that maintains the membrane's shape and stiffness through interconnecting spectrin and actin. hSTOM is a member of the protein family that possesses a single stomatin/prohibitin/flotillin/HflK (SPFH) domain at the center of the molecule. Although SPFH domain proteins are widely distributed from archaea to mammals, the detailed function of the domain remains unclear. In this study, we first determined the solution structure of the SPFH domain of hSTOM (hSTOM(SPFH)) via NMR. The solution structure of hSTOM(SPFH) is essentially identical to the already reported crystal structure of the STOM SPFH domain (mSTOM(SPFH)) of mice, except for the existence of a small hydrophilic pocket on the surface. We identified this pocket as a phosphate-binding site by comparing its NMR spectra with and without phosphate ions. Meanwhile, during the conventional process of protein NMR analysis, we eventually discovered that hSTOM(SPFH) formed a unique solid material after lyophilization. This lyophilized hSTOM(SPFH) sample was moderately slowly dissolved in a physiological buffer. Interestingly, it was resistant to dissolution against the phosphate buffer. We then found that the lyophilized hSTOM(SPFH) formed a fibril-like assembly under electron microscopy. Finally, we succeeded in reproducing this fibril-like assembly of hSTOM(SPFH) using a centrifugal ultrafiltration device, thus demonstrating that the increased protein concentration may promote self-assembly of hSTOM(SPFH) into fibril forms. Our observations may help understand the molecular function of the SPFH domain and its involvement in protein oligomerization as a component of the membrane skeleton. (245 words).

## Highlights

• Solution structure of human stomatin SPFH domain is determined.
• A cryptic phosphate-binding pocket was identified.
• Stomatin SPFH domain can form a fibril-like assembly at a high concentration.
• Phosphate ions promote formation of the fibril-like assembly.

## Graphical abstract

**Figure:**
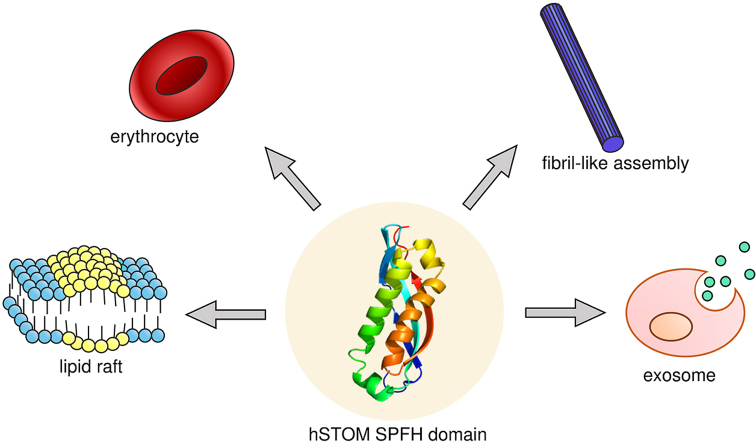

## 1 Introduction

**Fig. 1:**
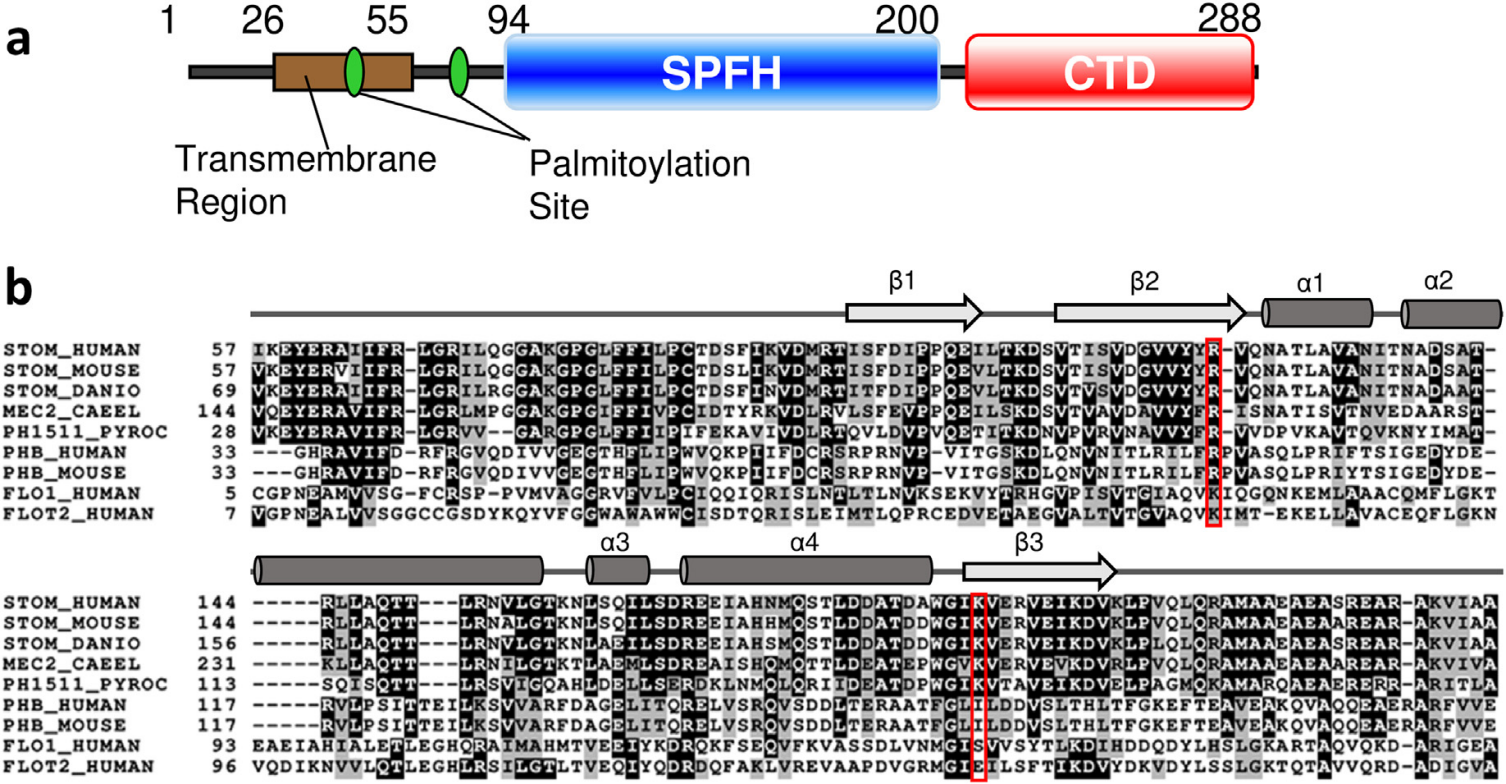
Architecture of human stomatin and sequence alignment of the SPFH domain. **a.** Domain arrangements, positions of the post-translational modifications, and predicted coiled-coil region for hSTOM(SPFH). **b.** Multiple sequence alignment of the core region of selected SPFH domain-containing proteins. The secondary structural elements of hSTOM(SPFH) are shown at the top of the diagram. Arrows and cylinders represent helices and strands, respectively. Protein names and the UniProt entries are as follows: STOM_HUMAN (human, Stomatin, P27105), STOM_MOUSE (mouse, Stomatin, P54116), STOM_DANIO (Zebrafish, Stomatin, O93423), MEC2_CAEEL (*C. elegans*, Mechanosensory protein 2, Q27433), PHB_HUMAN (human, Prohibitin-1, P35232), PHB_MOUSE (mouse, Prohibitin-1, P67778), FLO1_HUMAN (human, flotillin 1, O75955), FLOT2_HUMAN (human, flotillin 2, Q14254).

Stomatin (STOM), a band 7.2 protein, is one of the major components of the membrane skeleton in erythrocytes (Green et al., 2004). STOM is believed to maintain the membrane structure via interconnecting spectrin and actin and to reduce leakage of monovalent cations from red blood cells by regulating membrane stiffness. Inherited anemia, called stomatocytosis, results from a genetic defect in the STOM gene (Delaunay, 2004; Green et al., 2004; Stewart et al., 1993). STOM is also expressed in many vertebrate tissues and cell lines (Green et al., 2004). In cell types other than red blood cells, STOM has been reported to localize at the lipid raft microdomain (Browman et al., 2007; Dermine et al., 2001; Morrow and Parton, 2005; Salzer and Prohaska, 2001; Tavernarakis et al., 1999). STOM is a member of the protein family that possesses a single stomatin/prohibitin/flotillin/HflK (SPFH) domain at the center of the molecule. Human stomatin (hSTOM), a 288-residue protein, has a transmembrane region at the N-terminus and a single SPFH domain, followed by a coiled-coil region (Fig. 1a). STOM binds and regulates acid-sensing, non-voltage gated Na^+^-channels (ASICs) (Price et al., 2004), and GLUT-1 glucose transporter (Zhang et al., 1999). Although a long history of physiological investigations has suggested STOM's influence in the regulation of membrane itself as well as membrane proteins such as channels and transporters, the underlying molecular functions of the SPFH domain remain unclear.

The SPFH domain commonly contains three α-helices and three β-strands forming one antiparallel β-sheet (Yokoyama et al., 2008). It adopts a two-layer structure of an α-helical sub-domain and a β-sheet sub-domain connected by two linker regions at one side of the domain. The SPFH domain is evolutionarily conserved in eukaryotes, such as yeasts, plants, and mammals, as well as in bacteria and archaea (Tavernarakis et al., 1999). One of the proposed molecular functions of the SPFH domain is its potency in cholesterol binding (Huber et al., 2006). For example, binding between the SPFH domain in podocin and cholesterol has been reported. Nevertheless, an expanded hypothesis––that the common molecular function of the SPFH domain is cholesterol binding––is not likely because SPFH domain proteins are also found in bacteria and archaea, which lack the biosynthetic pathway of cholesterol (Green et al., 2004; Pearson et al., 2003). In contrast, oligomer formation is another possible molecular function of the SPFH domain (Kuwahara et al., 2009). In our previous study, we succeeded in determining the solution structure of the SPFH domain alone from *Pyrococcus horikoshii* PH0470. In addition, we reported that the SPFH domain of the PH0470 protein formed various oligomers and a multimer even without the coiled-coil region at the C-terminal end of the domain (Kuwahara et al., 2009). In this context, we started a solution NMR analysis of the SPFH domain of hSTOM [residues 94–202, hSTOM(SPFH)] and have already reported its near-complete NMR signal assignment (Tsuruta et al., 2012). hSTOM(SPFH) shows a striking sequence conservation of greater than 96% among all mammalian STOMs (Fig. 1b). The crystal structure of the SPFH domain of mouse STOM (mSTOM(SPFH)) has been reported (Brand et al., 2012). hSTOM(SPFH) may likely have a potency to form either oligomer or multimer, since a full-length STOM is known to oligomerize into 9–12 mers (Snyers et al., 1998). This hypothesis was partially proven by the crystal structure of the close paralogs of PH0470—the SPFH domain of PH1511—that formed a symmetrical trimer in the crystal (Yokoyama et al., 2008).

In this study, we focused on the potency of oligomer/multimer formation of hSTOM(SPFH) by various biophysical methods, including solution NMR. First, we succeeded in determining the solution structure of hSTOM(SPFH) and found a cryptic phosphate-binding pocket on the molecular surface. During the process of structure determination, we eventually found that the lyophilized sample of hSTOM(SPFH) was not readily soluble against phosphate buffer containing D_2_O. The lyophilized sample seemed to form a unique, slowly solubilizing solid material. Therefore, we analyzed the lyophilized hSTOM(SPFH) material by electron microscopy, and found that the sample formed a fibril-like assembly. This finding of the novel fibril-like hSTOM(SPFH) assembly may help understand the molecular function of the SPFH domain and its influence in a physiological role as a membrane skeleton component.

## 2 Materials and methods

### 2.1 Protein expression and purification

The preparation of hSTOM(SPFH) and its alanine-substituted mutants were performed as described previously (Tsuruta et al., 2012). The plasmid vectors carrying the R125A and K188A mutants of GST-tagged hSTOM(SPFH) were prepared according to a standard PCR mutagenesis method using the QuikChange™ site-directed mutagenesis kit (Stratagene, La Jolla, CA, USA). Briefly, the GST-tagged hSTOM(SPFH) (wild type and the mutants) was expressed in *Escherichia coli* BL21 (*DE3*) grown in M9 minimal medium containing [^15^N]-NH_4_Cl or LB medium under isopropyl-β-_D_-1-thiogalactopyranoside (IPTG) induction. The supernatant of the sonicated cells was purified using DEAE Sepharose (Cytiva, Marlborough, MA, USA) and Glutathione Sepharose 4 Fast Flow (Cytiva). After the cleavage of the GST-tag using thrombin (Wako) at 25 ​°C, the protein was further purified using size exclusion chromatography with a Superdex 75 HR 26/60 column (Cytiva) equilibrated with 50 ​mM Tris/HCl buffer (pH 7.5) containing 150 ​mM NaCl. The purified proteins were concentrated and dialyzed with 50 ​mM sodium phosphate buffer (pH 6.0) containing 100 ​mM NaCl or 50 ​mM bis-Tris/HCl (pH 6.0) containing 100 ​mM NaCl, depending on the experiment.

### 2.2 NMR experiments

The NMR experiments for solution structure determination were performed on a Bruker Avance III (900 ​MHz) NMR spectrometer (Bruker, Karlsruhe, Germany) equipped with a cryogenic triple-resonance probe. For the amide ^1^H chemical shifts temperature coefficients (^1^H-CSTC) experiments, 100 ​μM of monomeric ^15^N-hSTOM(SPFH) was dissolved in a buffer containing 50 ​mM sodium phosphate buffer (pH 6.4) containing 100 ​mM NaCl and 5% (v/v) D_2_O–95% H_2_O. Using this sample, ^1^H–^15^N HSQC spectra were acquired at four different temperatures (288 ​K, 293 ​K, 298 ​K, 303 ​K). The changes in the ^1^H chemical shifts according to temperature change (^1^H-CSTC) were calculated through a linear regression analysis (Cierpicki et al., 2002; Cierpicki and Otlewski, 2001). Residues with a ^1^H-CSTC greater than the threshold of −4.6 ​ppb/K were assumed to be hydrogen bonded, while those with one lower than the threshold were assumed to be not hydrogen bonded (Cierpicki and Otlewski, 2001). The hydrogen bond partners were determined based on the analysis of 11 independent monomer conformations of mSTOM(SPFH) deposited in PDB (4FVF, 4FVG, 4FVJ) (Brand et al., 2012). Chemical shift assignments of hSTOM(SPFH) have already been published (Tsuruta et al., 2012). All of the nuclear Overhauser effect (NOE) restriction constraints, dihedral angle constraints, and hydrogen bond restrictions were integrated and subjected to the CYANA (ver 2.1) structure calculation (Güntert, 2003, 2004). The top 20 structures calculated from 200 initial structures by CYANA were then subjected to the refinement process using CNS (ver 1.2) (Brunger, 2007). The conventional simulated annealing protocol was applied for the final refinement. All figures were prepared using PyMOL (open source version, http://www.pymol.org). The atomic coordinates of the 20 best hSTOM(SPFH) NMR structures have been recorded in the Protein Data Bank under accession code 7WH3 (http://www.pdbj.org/).

For the phosphate-binding experiments, 100 ​μM of ^15^N-hSTOM(SPFH) was dissolved in a solution containing 50 ​mM sodium phosphate buffer (pH 6.0) containing 100 ​mM NaCl and 5% (v/v) D_2_O or 50 ​mM bis-Tris/HCl (pH 6.0) containing 100 ​mM NaCl and 5% (v/v) D_2_O. Then, ^1^H–^15^N HSQC spectra were obtained. The normalized chemical shift change (Δδ_H,N_) was calculated with the following equation (1):

(1) $$\text{Δ}\delta_{\text{H},\text{N}}=\sqrt{{\left(\text{Δ}\delta_{\text{H}}\right)}^{2}+{\left(\text{Δ}\delta_{\text{N}}/5\right)}^{2}}$$

where Δδ_H_ and Δδ_N_ are the chemical shift differences in the presence and absence of the phosphate ions, respectively.

### 2.3 Light scattering experiment

The precipitated or insoluble fraction of hSTOM(SPFH) was monitored by measuring the light scattering at 320 ​nm using a fluorophotometer F-7000 (Hitachi) with a 10 ​mm four-face polished quartz square cuvette. Lyophilized hSTOM(SPFH) was suspended in 50 ​mM sodium phosphate buffer (pH 6.0) containing 100 ​mM NaCl or 50 ​mM bis-Tris/HCl (pH 6.0) containing 100 ​mM NaCl to a final concentration of 400 ​μM. All samples were processed by sonication for 5 ​s after gentle pipetting. Both excitation and measurement wavelengths were 320 ​nm. For all samples, measurements were performed three times.

### 2.4 Electron microscopy

hSTOM(SPFH) dissolved in 50 ​mM sodium phosphate buffer (pH 6.0) containing 100 ​mM NaCl was lyophilized and then was dissolved with distilled water to a final concentration of 100 ​μM followed by sonication for 5 ​s. Non-lyophilized hSTOM(SPFH) was dialyzed in 50 ​mM sodium phosphate buffer (pH 6.0) containing 100 ​mM NaCl, and then diluted to 100 ​μM in the same buffer followed by sonication for 5 ​s. For the observation of fibrils formed by ultrafiltration, 100 ​μM STOM(SPFH) in 50 ​mM sodium phosphate buffer (pH 6.0) containing 100 ​mM NaCl was concentrated with Nanosep 3 ​K (PALL, Port Washington, NY). The sample aliquot was placed on a glow-discharged, carbon-coated copper grid and negatively stained with 2% uranyl acetate. A JEM-1010 microscope (JEOL, Tokyo, Japan) was used for observation.

### 2.5 Nanoparticle tracking analysis (NTA)

hSTOM(SPFH) samples after lyophilization were well suspended in distilled water to a concentration of 400 ​μM. The sample was then diluted 100-fold with 50 ​mM sodium phosphate buffer (pH 6.0) containing 100 ​mM NaCl to a final concentration of 4 ​μM. Then the samples were analyzed by Nanosight NS300 (Malvern, UK) according to the manufacturer's instruction at room temperature. The protein solution was collected in a 1 ​mL disposable syringe and measured while pumping with a syringe pump. The software NTA 2.3 was used for video capture and data analysis. The results of the 5 measurements were averaged and the histogram was created.

## 3 Results

### 3.1 Structural determination of the hSTOM SPFH domain

**Fig. 2:**
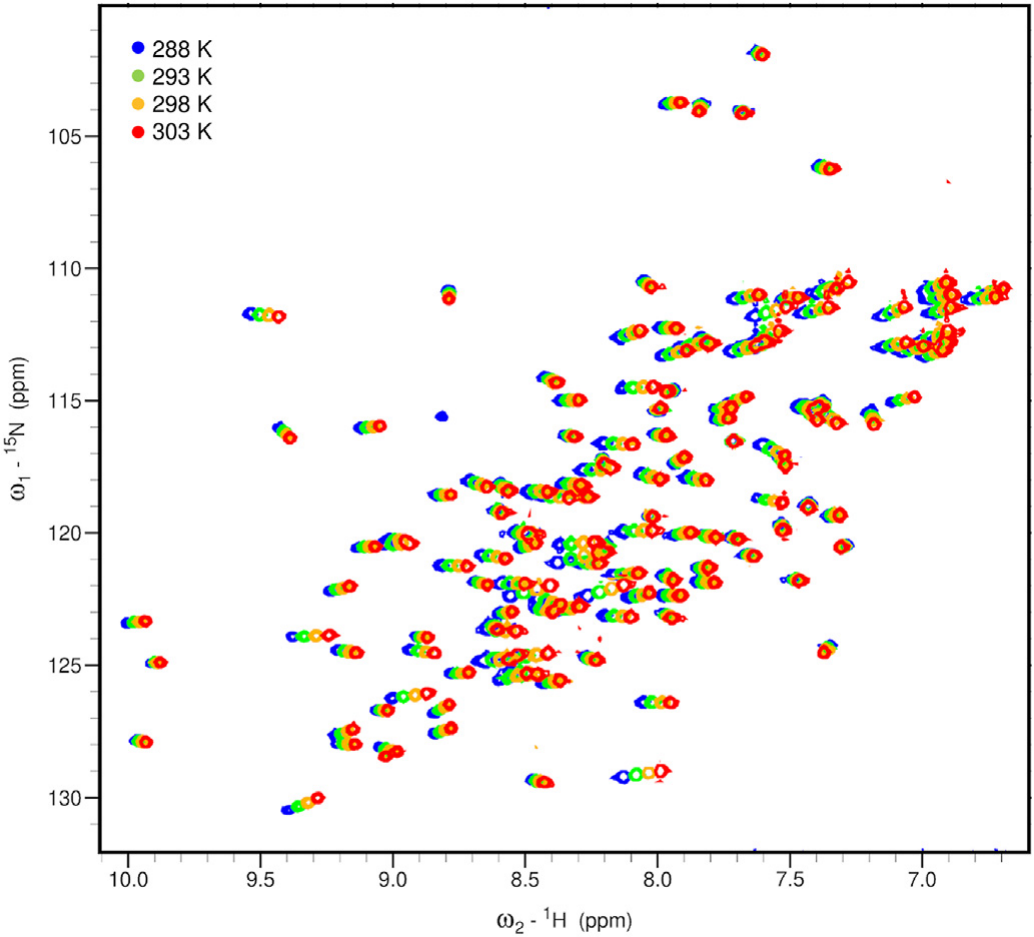
HSQC spectra of the hSTOM(SPFH) recorded at four temperatures. Signals of 288, 293, 298, and 303 ​K are shown in blue, green, yellow, and red, respectively.

We first attempted to determine the solution structure of hSTOM(SPFH) by employing standard solution NMR techniques followed by CYANA calculation (Güntert, 2004; Yamazaki et al., 1994). In order to obtain information on intra-molecular hydrogen bonds, we performed a conventional H/D exchange experiment in which the buffered hSTOM(SPFH) sample in a H_2_O based solvent was lyophilized and dissolved in D_2_O, and the decreasing NH signal intensities were monitored. However, this attempt was eventually hampered by an unexpected characteristic of the sample; the lyophilized hSTOM(SPFH) did not dissolve immediately upon the mMaddition of D_2_O and dissolved very slowly. This sample was completely dissolved only after three days, and it seemed to be an intact form, which was confirmed by the CD and NMR spectra (data not shown). The lack of hydrogen bond constraints during the CYANA calculation hindered the determination of a high-quality solution structure (data not shown), although more than 95% of ^1^H signals were assigned (Tsuruta et al., 2012). Therefore, we employed the ^1^H-CSTC experiment as a method for estimating hydrogen bond-forming residues. ^1^H-CSTC and the formation of intramolecular hydrogen bonds have been reported to be in good agreement (Cierpicki and Otlewski, 2001). We used a threshold value of −4.6 ​ppb/K to estimate the existence of intramolecular hydrogen bonds. Consequently, 64 of 113 residues were judged to form intramolecular hydrogen bonds (Fig. 2, Table 1). We searched for hydrogen-bonding partners among 11 individual structures from the PDB entries of mSTOM(SPFH) crystal structures as models and estimated 104 hydrogen bond constraints corresponding to 52 residues that form hydrogen bonds among the main chains. All of the experimental NMR constraints derived from the NOE peak list, the dihedral angle constraints, and the hydrogen bond constraints were then subjected to CYANA (ver. 2.1). After several adjustments to noise signals in the NOESY peak lists, the final CYANA results were further refined using CNS (ver 1.2). The 20 structures with the lowest energy were accepted as the final structures. We succeeded in determining the solution structure of hSTOM(SPFH) with a reasonable quality (Supplementary Fig. S1, Ramachandran plot). Experimental restraints and statistics of structure determination were summarized in Table 2. The root mean square deviation (r. m. s. d.) of the backbone atoms and that of the heavy side chain atoms are 0.23 Å and 1.41 Å, respectively.

**Table 1:** Hydrogen bonds in hSTOM(SPFH) determined by ^1^H-CSTC experiments.

^1^H-CSTC		Hydrogen bonds in crystal structures^a^	
> −4.6 ​ppb/K	63	With intramolecular	79
< −4.6 ​ppb/K	38	With solvent	34
others^b^	9		
prolines	3		
Total 113			

a The structure of the 11 monomers in the crystal structure of mouse stomatin in PDB was used as a reference.

b Those could not be identified due to overlapping or containing threshold value in the error range.

**Table 2:** Experimental Restraints and Statistics for 20 Structures of hSTOM(SPFH).

**Distance restraints**	
Total number of restrains	2148
Intra-residual	316
Sequential restraints [ | i-j | ​= ​1 ]	666
Medium-range restraints [ 1 ​< ​| i-j | ​≤ ​4 ]	569
Long-range restraints [ | i-j | ​> ​4 ]	597
Dihedral angle restraints	111
Phi/Psi/Chi	55/56/0
Hydrogen bond restraints	55 ​× ​2
**Statistics used for and obtained from the structure calculations**	
Final Statistics (20/100)	
Cutoffs: Distance (0.3 ​Å) and Angle (3.0 deg.)	
Maximum CYANA target function	4.60
Maximum violations in CYANA calculation	
Distance violation (Å)	0.81
Angle violation (deg.)	7.59
Maximum CNS Overall Energy	60.57
Coordinate precision (residues 98–195)^a^	
Backbone r. m. s. d. (Å)	0.23 ​± ​0.07
Heavy atoms r. m. s. d. (Å)	1.41 ​± ​0.50
Ramachandran plot statistics (%)	
Residues in most favored regions	89.6
Residues in additionally allowed regions	10.4
Residues in generously allowed regions	0.0
Residues in disallowed regions	0.0

a Residue numbers correspond to those in the full length human stomatin (accession: AAH10703).

**Fig. 3:**
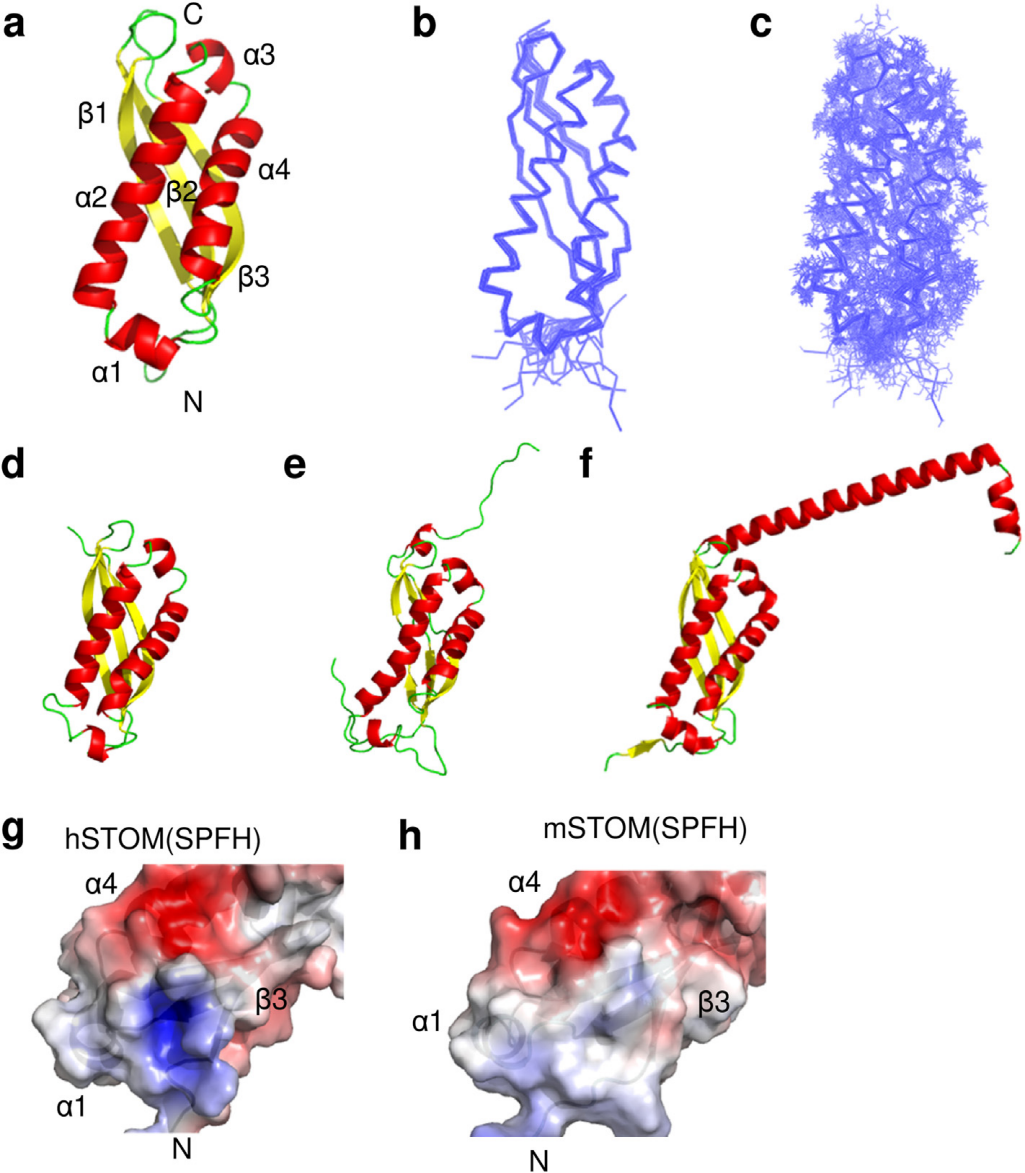
Solution structure of hSTOM(SPFH). **a.** Solution structure of hSTOM(SPFH); α-helices are shown in red, β-strands in yellow. **b.** The best fit superposition of the 10 structures with the fewest structural violations. **c.** Same as b, and the sidechains are represented. **d.** Ribbon diagram of mSTOM(SPFH) (PDB ID: 4FVG). **e.** Human flotillin 2 (PDB ID: 1WIN). f. *P. horikoshii* PH1511 SPFH (PDB ID: 3BK6). **g, h.** Comparison of the positively charged surface of hSTOM(SPFH) (solution, from this study) and mSTOM(SPFH) (crystal).

We confirmed that the hSTOM(SPFH) adopts to an *α*/*β*-fold structure comprising four α-helices (*α*1 (residues 129–135), *α*2 (residues 139–157), *α*3 (residues 160–165), and *α*4 (residues 167–185)) and three β-strands (β1 (residues 98–110), β2 (residues 114–126), and β3 (residues 187–198)) (Fig. 3a). The superposition of the calculated solution structures converged effectively (Fig. 3b and c) and was primarily identical to the crystal structures of the mSTOM(SPFH) (Fig. 3d). For example, there are 11 independent monomer coordinates found in the three deposited PDB entries (4FVF, 4FVG, and 4FVJ) (Brand et al., 2012). The backbone r. m. s. d. of these crystal structures was 0.75 ​Å (Supplementary Fig. S2). This suggests that the accuracy of our solution structure of hSTOM(SPFH) is acceptable compared to the structural fluctuation in the crystals. The arrangement of the secondary structure is also similar to the other SPFH domains of the related proteins: flotillin-2 and *Pyrococcus* stomatin PH1511 (Fig. 3e ​and ​f). However, when superimposing the represented structure of hSTOM(SPFH) with that of mSTOM(SPFH), we found a substantial structural difference (Fig. 3g ​and ​h and , see below). We found a small, positively charged pocket around the C-terminal end of α1, which was absent in the mSTOM(SPFH) crystal structure.

### 3.2 Characterization of the lyophilized hSTOM(SPFH) sample in aqueous buffers

**Fig. 4:**
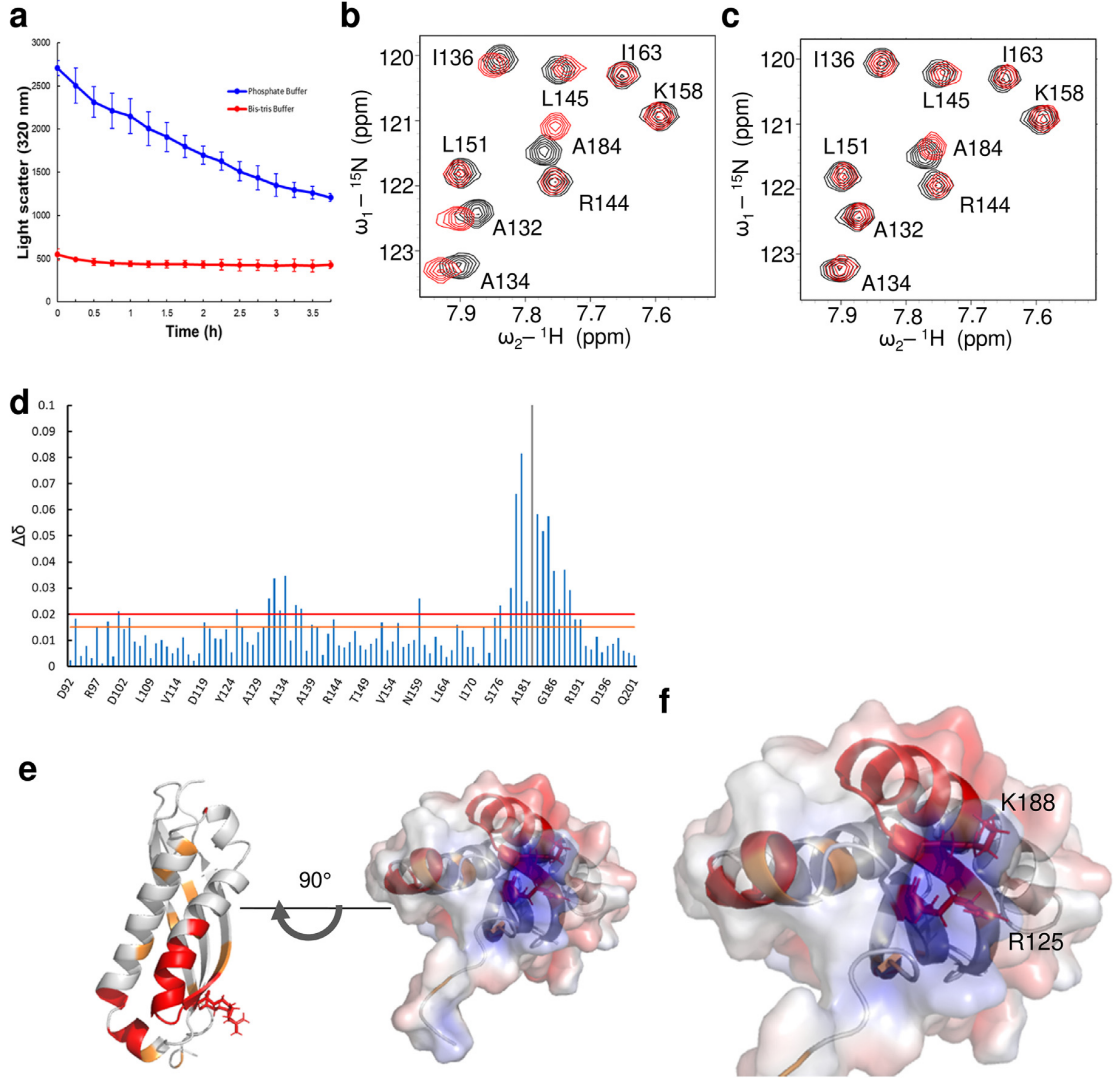
hSTOM(SPFH) directly interacted with phosphate ions. **a.** Time-dependent changes in light scattering of the lyophilized hSTOM(SPFH) sample prepared in the two different buffer conditions. Navy represents the results of the phosphate buffer [400 ​μM hSTOM(SPFH), 50 ​mM sodium phosphate, 100 ​mM NaCl, pH 6.0] and red represents the result of the Bis-Tris buffer. **b.** Overlay of HSQC spectra of hSTOM(SPFH) under phosphate buffer (black) or bis-Tris buffer (red). The peaks are shifted for some residues. **c.** Same as b, phosphate buffer (black) or bis-Tris buffer supplemented with sodium phosphate (red). **d.** Normalized chemical shift differences of amide signals between the signal in the phosphate-based buffer and that in the Bis-Tris buffer without phosphate ion. The lines are a thresholds to discriminate the residues of remarkable chemical shift perturbation (CSP) (orange: Δδ ​≥ ​0.015, red: Δδ ​≥ ​0.020). **e.** Mapping of the residues of remarkable CSPs upon phosphate addition (orange: Δδ ​≥ ​0.015, red: Δδ ​≥ ​0.020). **f.** A close-up view of a cryptic phosphate-binding site. The sidechains of R125 and K188 are marked.

We eventually discovered that the lyophilized hSTOM(SPFH) NMR sample has a unique character and is resistant to re-dissolution in the aqueous buffer. This phenomenon was repeatedly observed and reproduced. We then characterized the process of dissolution of the lyophilized material against various buffers by light scattering. The hSTOM(SPFH) was dialyzed against either Bis-Tris buffer (50 ​mM Bis-Tris/HCl buffer (pH 6.0) containing 100 ​mM NaCl) or phosphate buffer (50 ​mM sodium phosphate buffer (pH 6.0) containing 100 ​mM NaCl) and then lyophilized. Then, each sample was well suspended in distilled water, and the time-dependent change in scattered light was monitored (Fig. 4a).

For the phosphate buffer sample, the initial intensity of light scattering of the sample immediately after suspension was high, and the intensity slowly decreased over hours (Fig. 4a). Subsequently, the residual basal scattered light remains constant over days. On the other hand, when the sample was suspended in the Bis-Tris buffer, most parts of the aggregate immediately dissolved, and the scattered light was low. The results indicated that some parts of the lyophilized hSTOM(SPFH) material are more resistant to being solubilized in the phosphate-based buffer than the buffer without phosphate ions. We further found that the buffers containing oxyanions other than phosphate ion, including sulfate, nitrate, and bicarbonate, are incapable to promote aggregation similar to Bis-Tris buffer (Supplementary Fig. S3).

### 3.3 A cryptic phosphate binding site of hSTOM(SPFH)

We then examined whether phosphate ion(s) can directly interact with the molecular surface of hSTOM(SPFH) by NMR experiments. A comparison of ^1^H–^15^N HSQC spectra in the phosphate buffer and Bis-Tris buffer revealed chemical shift changes in more than 35 NH signals (Fig. 4b and Supplementary Fig. S4). We hypothesized that these signal differences were due to the presence and absence of phosphate ions, and we added sodium phosphate to the sample prepared in the Bis-Tris buffer to final concentration of 50 ​mM, following which ^1^H–^15^N HSQC spectra were measured (Fig. 4c). As expected, the obtained HSQC spectra were similar to that of the phosphate buffer, and the chemical shift differences of these two spectra were negligible (Fig. 4c). Then, the residues with significant chemical shift changes between the phosphate and Bis-Tris buffers were identified and mapped on the solution structure determined in this study (Fig. 4d–f). Obviously, these residues formed a cluster around the helices α1, α4, and the N-terminal end of the strand β2. It should be noted that a small, positively charged pocket was found at the center of the cluster close to α4, β2, and β3 (Fig. 4e). This pocket is cryptic and unique to the solution structure of hSTOM(SPFH), and it was absent in the crystal structure(s) of mSTOM(SPFH) (Fig. 3g and h), probably because of the difference of the buffer condition of the structure determination (either in the presence or the absence of the phosphate ion).

### 3.4 Identification of the residues responsible for phosphate binding

**Fig. 5:**
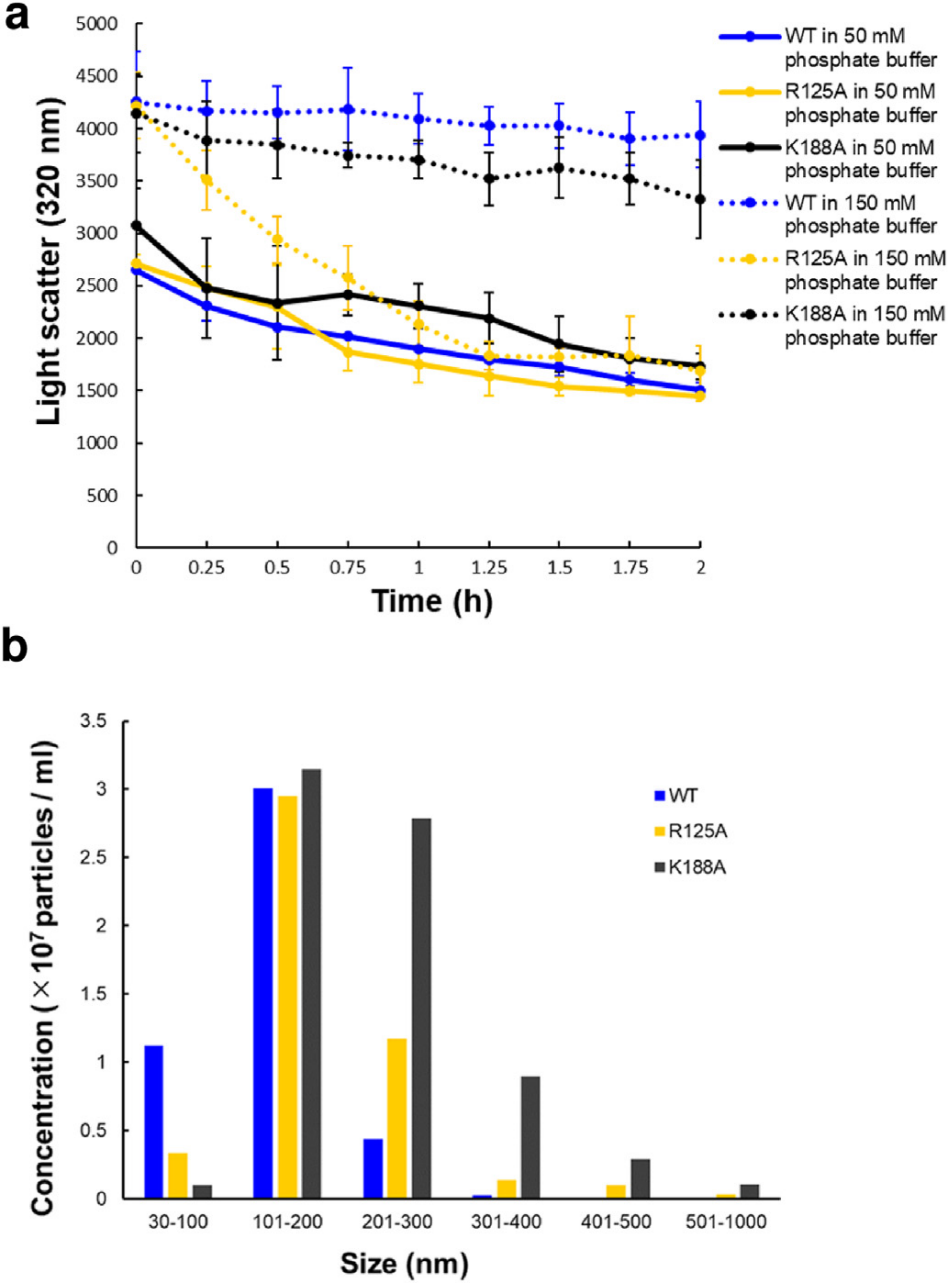
Effect of alanine substitution of R125 and K188 on the lyophilized solid hSTOM(SPFH) aggregates in the phosphate buffer. **a.** Time-dependent decrease in light scattering. Navy, wild type; yellow, R125A; black, K188A. The solid line indicates the result of 50 ​mM sodium phosphate, and dashed line is the result of 150 ​mM sodium phosphate. **b.** Particle size profile of the lyophilized solid hSTOM(SPFH) aggregates (wild type, R125A, and K188A) suspended in the phosphate buffer by NanoSight. The lower detection limit of the instrument is 30 ​nm.

We then attempted to identify the critical residues of hSTOM(SPFH) for phosphate ion binding by amino acid substitution experiments. First, we focused on the two positively charged residues—R125 and K188—that are located at the center of the phosphate-binding pocket (Fig. 4g and f). Note that these residues are well conserved over the vertebrate stomatins (Fig. 1b). To examine the relevance of these residues, we prepared two alanine-substituted mutants, hSTOM(SPFH)-[R125A] and [K188A]. As shown previously, the binding of phosphate ions to the phosphate-binding pocket markedly reduces the solubility of the lyophilized solid material of hSTOM(SPFH). Thus, we employed the same light-scattering experiments to evaluate the hSTOM(SPFH) mutants (Fig. 5a). As shown in Fig. 5a, we observed no difference between the wild type (WT) and the mutants under a low phosphate buffer condition. However, when they were redissolved in the buffer containing 200 ​mM sodium phosphate, hSTOM(SPFH)[R125A] showed a faster dissolution profile compared to WT and K188A, which were resistant to dissolution for a long time. This result demonstrated that R125 in hSTOM(SPFH) is a key residue for phosphate ion binding and regulating the stability of the hSTOM(SPFH) aggregate.

We further analyzed the time-dependent changes in the particle sizes of the lyophilized wt and mutant hSTOM(SPFH) materials after suspension into the phosphate-based buffer using the nanoparticle tracking analyzer NanoSight NS300 (Malvern Panatycal Ltd., Malvern, UK) (Fig. 5b). Interestingly, both mutants, R125A and K188A, showed particle size profiles different to the wild type. The K188A material contained an increased number of larger (>300 ​nm) particles, whereas the wild type hSTOM(SPFH) was mainly dispersed as smaller (<300 ​nm) particles. R125A was in between these. We concluded that genetically conserved K188 may also partly contribute to phosphate ion binding, although its contribution to the poor solubility of the lyophilized material is small.

### 3.5 The hSTOM(SPFH) aggregates contain fibril-like self-assembly

**Fig. 6:**
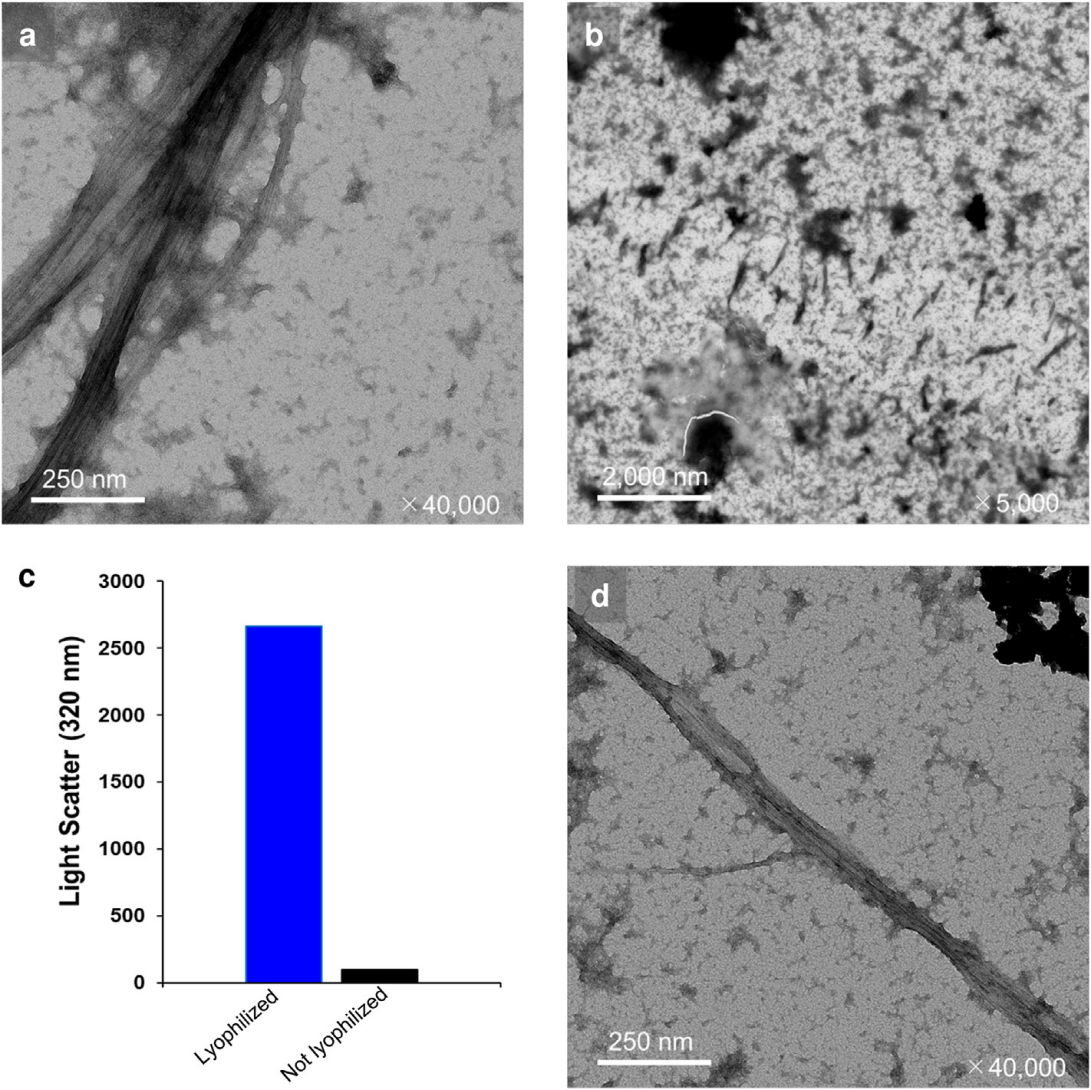
Electron microscopic observation of the fibril-like assembly of hSTOM(SPFH). **a.** Fibril-like assembly of hSTOM(SPFH). hSTOM(SPFH) remaining in distilled water after lyophilization, as observed by electron microscopy. Buffer conditions were 50 ​mM sodium phosphate, 100 ​mM NaCl, pH 6.0. Magnification was 40,000x. **b.** Non-lyophilized hSTOM(SPFH). Electron microscopy of hSTOM(SPFH) without lyophilization. Buffer conditions were 50 ​mM sodium phosphate, 100 ​mM NaCl, pH 6.0. Magnification was x5,000. **c.** Amount of remaining dissolved protein in lyophilized and non-lyophilized samples. Blue is the freeze-dried sample and black is the non-freeze-dried sample. Both are added to 50 ​mM sodium phosphate, 100 ​mM NaCl, pH 6.0. **d.** Fibril-like assembly produced by centrifugal ultrafiltration. The aggregates found on the membrane of the centrifugal ultrafiltration device was observed by TEM. The buffer conditions were 50 ​mM sodium phosphate, 100 ​mM NaCl, pH 6.0.

In general, many well-behaved soluble protein samples can be easily lyophilized, and then immediately re-dissolved in an appropriate buffer. On the other hand, some aggregation-prone or unstable proteins are not suitable for lyophilization, and they tend to denature after re-dissolution. Since the hSTOM(SPFH) sample did not show such characteristics, we examined the fine structure of the lyophilized solid material of hSTOM(SPFH) via negative-stain transmission electron microscopy (TEM). We found a unique fibril-like structure (Fig. 6a). This unique fibril-like structure was specific to its preparation (i.e., lyophilization). We did not observe any fibrils in the sample without lyophilization, which was first concentrated by a centrifugal device in Bis-Tris buffer and then dialyzed to the same phosphate buffer (Fig. 6b). The approximate number of observed multimers was estimated and compared (Fig. 6c). We assumed that the increase in the local concentration of hSTOM(SPFH) during the lyophilization-dissolving process may trigger the formation of the fibril-like self-assembly. Subsequently, we tried to reproduce similar fibril formation using a different experimental procedure. During the conventional laboratory protocol of preparing NMR samples, we eventually experienced that the solution of hSTOM(SPFH) in 50 ​mM phosphate buffer (pH 6.0) became turbid on the membrane of a centrifugal ultrafiltration device. We picked up the opaque precipitate and examined it via negative stain TEM (Fig. 6d). As expected, the similar fibril-like self-assembly of hSTOM(SPFH) was successfully reproduced by centrifugal ultrafiltration. This self-assembly on the filtration membrane was also promoted by phosphate buffer rather than Bis-Tris buffer (Supplementary Fig. S5).

## 4 Discussion

In this study, we demonstrated that the solution structure of hSTOM(SPFH) was essentially identical to that of the crystal structure(s) of mSTOM(SPFH). Notably, mSTOM(SPFH) was reported as forming a banana-like dimer in the crystal and was in a monomer–dimer equilibrium at the concentration of μM range (Brand et al., 2012). In that dimer, two protomers were stabilized by the hydrogen bonds between V197 and L199. In contrast, in the solution structure of hSTOM(SPFH), the corresponding hydrogen bonds were not detected by ^1^H-CSTC experiments, even though the experiments were performed at 0.1 ​mM, a concentration high enough to detect the dimer species of the reported *K*_d_. Therefore, the current construct of hSTOM(SPFH) unlikely to form a dimer under the present measurement conditions. Moreover, we did not observe a putative hydrophobic pocket for accepting the other C-terminal peptide reported in the mSTOM(SPFH) crystal structure (Brand et al., 2012). This is due to the lack of the corresponding C-terminal Ile-Leu fragments in the hSTOM(SPFH) construct, which fit into the hydrophobic pocket in the crystal structure of mSTOM(SPFH). In contrast, the positively charged pocket observed in the solution structure of hSTOM(SPFH) was not observed in mSTOM(SPFH), since the buffer condition of the crystallization of mSTOM(SPFH) did not contain phosphate ion. In other words, a certain shift (∼3.4 ​Å) in the relative position of α1 was observed, and the orientation of the Q127 sidechain also changed in the opposite direction, compared to that of mSTOM(SPFH) in the presence of phosphate ions. All these structural changes lead to cryptic phosphate-binding pocket formation. In addition, we further confirmed phosphate ion, but not sulfate ion (which was present in the crystallization condition of mSTOM(SPFH)) did not promote forming dissolution-resistant aggregate after lyophilization (Supplementary Fig. S3).

We demonstrated the relevance of the phosphate ion(s) for promoting and stabilizing the fibril-like self-assembly of hSTOM(SPFH) (Fig. 4a). The HSQC spectrum shows that the chemical shifts of more than 35 residues have been changed (Supplementary Fig. S4). This is a significant number considering the size of the phosphate ions, suggesting a chemical environment changes.

The corresponding phosphate binding pocket appears cryptic and probably sensitive to the cytosolic environment. Although R125 and K188 were suggested to be key residues in phosphate binding, their contributions to fibril stabilization were not the same. Under the low-phosphate condition, the behavior of the fibril-like assembly of the wild type and the mutant SPFH domain was similar. For the formation of the fibril-like assembly during lyophilization, R125A and K188A showed a similar level of turbidity at the time point zero, suggesting either one is sufficient for promoting fibril formation (Fig. 5a). Subsequently, both mutants dissolved faster than the wild type; thus, these two residues contributed to stabilizing the fibril. R125 seems more responsible for phosphate-binding than K188, as R125A is less resistant to dissolving in a high-phosphate buffer. Notably, these residues are genetically conserved among mammalian and fish stomatins (Fig. 1b). In addition, these residues are conserved in nematode mechanosensory component mec-2 and archaeal stomatin homolog *Pyrococcus horikoshii* PH1511 with unknown functions.

We subsequently demonstrated the formation of a similar fibril-like assembly of hSTOM(SPFH) induced by the two experimental procedures, lyophilization and centrifugal ultrafiltration. Since the fibrils looked very similar under the negatively stained TEM, we concluded that the increase in the local concentration of hSTOM(SPFH) is a trigger for fibrilization. This fibril formation was achieved without the N-terminal membrane-spanning helix and the C-terminal domain containing the coiled-coil region. In addition, stomatin is an abundant protein in the erythrocyte membrane skeleton and localized to the membrane, and a full-length stomatin is reported to exist as 9–12 mers (Snyers et al., 1998). Thus, it is likely to form similar fibril-like structures, even *in vivo*.

The nature of the ring-shaped multimer formation of the other SPFH domain containing proteins is widely known. We previously reported the ring-like multimer with an approximate molecular weight of >600 k containing the SPFH-domain-only construct from the archaeal stomatin PH0470, a paralog of PH1511 (Kuwahara et al., 2009). Similar ring-shaped multimers have been reported in prohibitin-1/2 heterodimer super-complex (Tatsuta et al., 2005) and Cyanobacterium (*Synechocystis* sp.) Slr1128 (Boehm et al., 2009). The “shoulder” domain of the *Rattus norvegicus* major vault protein (RnMVP), a distant homolog of the SPFH domain, has been shown to form a rigid ring shape (Tanaka et al., 2009). More recently, the periplasmic region of FliL (FliL_Peri_) from *Vibrio alginolyticus*, a component of the flagellar motor complex, also showed a ring-like assembly in its crystal structure (Takekawa et al., 2019). Very recently, in HflC/K-FtsH complex, SPFH domains of HflC and HflK have been shown to form a 24-member ring (Ma et al., 2022). In the last two examples, the SPFH domains of the proteins directly contacted each other to coordinate ring-shaped multimers. This evidence suggests that a substantial part of the SPFH domain containing proteins functions as a scaffold by utilizing the ability of oligomerization/multimerization of the SPFH domain itself. Thus, we hypothesize that the SPFH domain in hSTOM can similarly contribute to multimerizing in full length hSTOM polypeptides. Unfortunately, we have not yet found any convenient staining methods for SPFH fibrils. For example, we failed to stain the SPFH fibrils with thioflavin-T, 1-Anilinonaphthalene-8-Sulfonic Acid, and Congo red dyes, all of which are frequently used to stain amyloid beta fibril and other cytoskeletons (data not shown). This lack of a specific stain for stomatin fibril hinders us from proving the existence of the fibril-like stomatin in erythrocytes. Nevertheless, our discovery of the fibril-like assembly of hSTOM(SPFH) and the experimental procedure (e.g., high phosphate condition) to reproduce the fibril material by lyophilization in vitro adds new insight in the research of stomatin. Thus, further studies are needed for the physiological relevance of the fibril-like self-assembly of hSTOM(SPFH).

## CRediT authorship contribution statement

**Koki Kataoka:** Investigation, Visualization, Writing – original draft. **Shota Suzuki:** Investigation, Visualization. **Takeshi Tenno:** Conceptualization, Investigation, Data curation, Resources. **Natsuko Goda:** Investigation, Resources. **Emi Hibino:** Investigation, Data curation, Formal analysis, Visualization, Writing – review & editing. **Atsunori Oshima:** Conceptualization, Data curation, Supervision. **Hidekazu Hiroaki:** Conceptualization, Data curation, Writing – original draft, Writing – review & editing, Supervision, Project administration, Funding acquisition.

## Declaration of competing interest

The authors declare that they have no known competing financial interests or personal relationships that could have appeared to influence the work reported in this paper.
