# Supplementary material for: A cryptic phosphate-binding pocket on the SPFH domain of human stomatin that regulates a novel fibril-like self-assembly
Source: Curr Res Struct Biol. 2022 May 18;4:158–66. doi: 10.1016/j.crstbi.2022.05.002 (PMC9157467; doi:10.1016/j.crstbi.2022.05.002)
Supplement: Multimedia component 1 [file mmc1.pdf]

Supplementary Figures

A cryptic phosphate-binding pocket on the SPFH domain of human stomatin that regulates a novel fibril-like self-assembly.

Koki Kataoka[1], Shota Suzuki[1], Takeshi Tenno[1,2], Natsuko Goda[1], Emi Hibino[1], Atsunori Oshima[1,3,4], and Hidekazu Hiroaki[1,2,3*].

[1]Laboratory of Structural Molecular Pharmacology, Graduate School of Pharmaceutical Sciences, Nagoya University, Furocho, Chikusa-ku, Nagoya, Aichi, Japan, 464-8601.

[2]BeCellBar LLC., Business Incubation Building, Nagoya University, Furocho, Chikusa-ku, Nagoya, Aichi, Japan, 464-8601.

[3]Cellular and Structural Physiology Institute (CeSPI), Nagoya University, Furocho, Chikusa-ku, Nagoya, Aichi, Japan, 464-8601.

[4]Institute for Glyco-core Research (iGCORE), Nagoya University, Furo-cho, Chikusa-ku, Nagoya 464-8601, Japan

Supplementary Figure S1

Ramachandran plot for 20 Structures of hSTOM(SPFH).

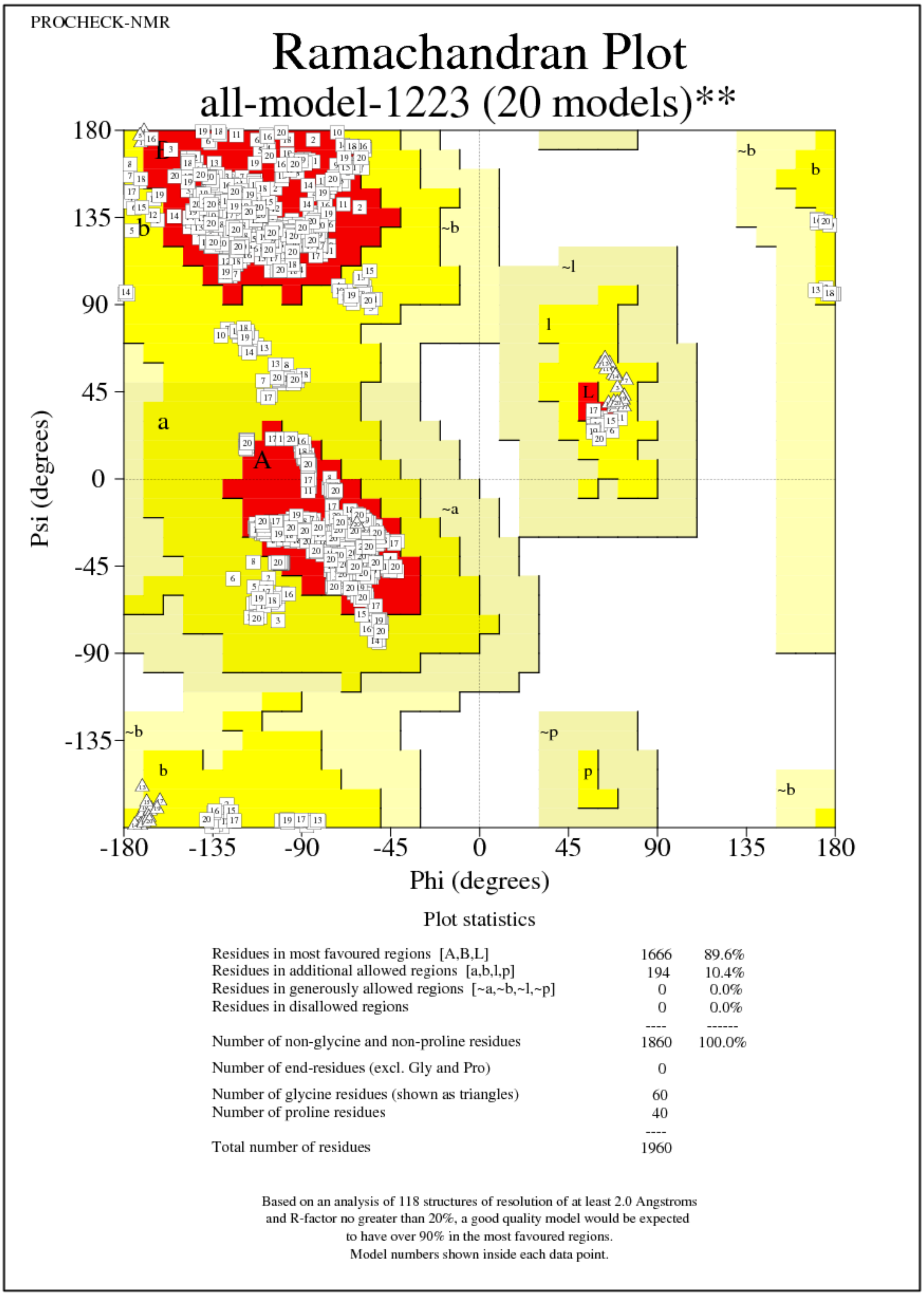

Supplementary Figure S2

Superposition of 11 unique crystal structure of mSTOM(SPFH) deposited in PDB (4FVF, 4FVG, 4FVJ). a, backbone representation; b, backbone with all sidechain heavy atoms.

a **b**

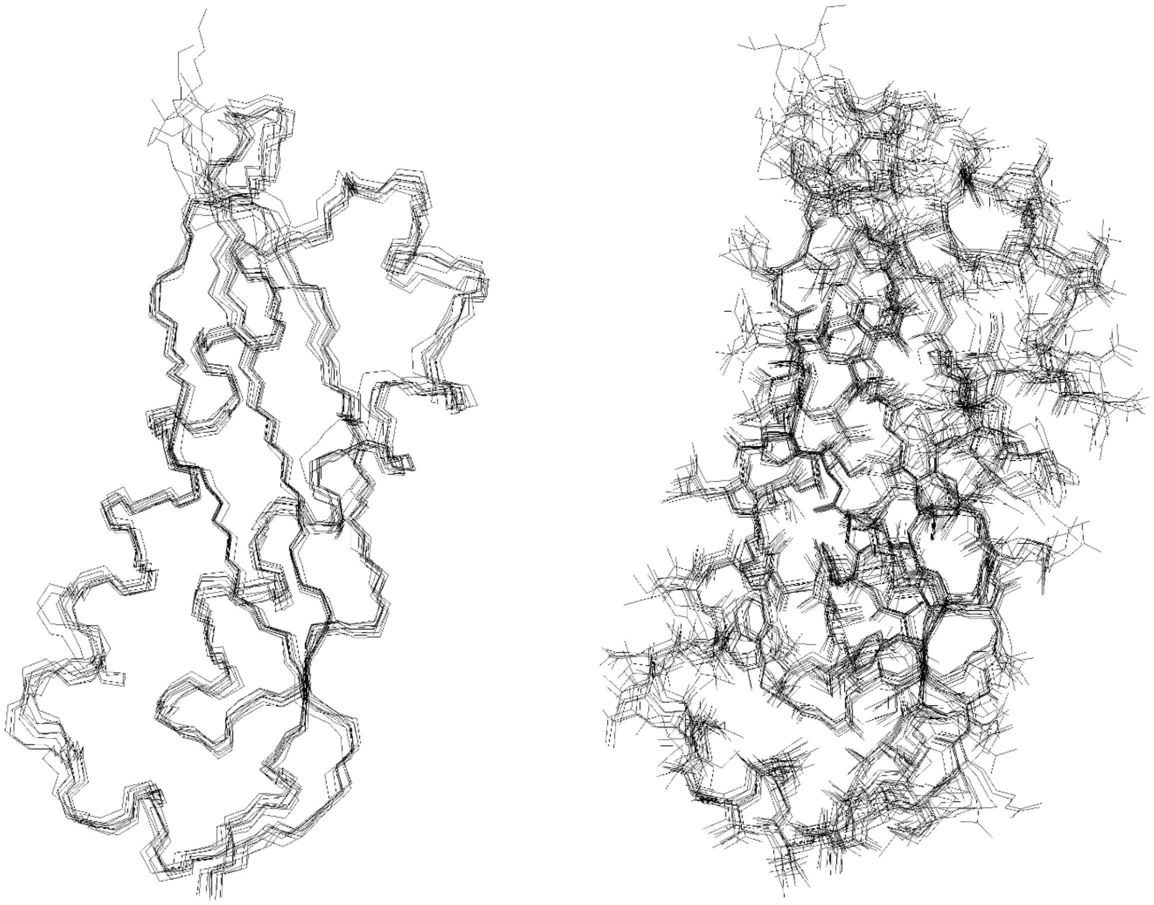

Supplementary Figure S3

Effect of different anions upon formation of dissolution-resistant solid material after lyophilization of hSTOM(SPFH). Light scattering of the lyophilized hSTOM(SPFH) in different buffers. hSTOM(SPFH) was lyophilized after dialysis into different buffers. The final 400 μM of hSTOM(SPFH) was lyophilized in buffer containing indicated ions, dissolved by deionized water, and the suspended solution was monitored by light scattering at 320 nm. [sodium phosphate] 50 mM sodium phosphate, 50 mM Bis-Tris/HCl (pH 6.0), 100 mM NaCl, [potassium phosphate] 50 mM potassium phosphate, 50 mM Bis-Tris/HCl (pH 6.0), 100 mM NaCl , [sodium sulfate] 50 mM sodium sulfate, 50 mM Bis-Tris/HCl (pH 6.0), 100 mM NaCl, [sodium nitrate] 50 mM sodium nitrate, 50 mM Bis-Tris/HCl (pH 6.0), 100 mM NaCl, [sodium hydrogen bicarbonate] 50 mM sodium hydrogen carbonate, 50 mM Bis-Tris/HCl (pH 6.0), 100 mM NaCl, [Bis-Tris/HCl] 50 mM Bis-Tris/HCl (pH 6.0), 100 mM NaCl (Bis-Tris/HCl), and [HEPES/NaOH] 100 mM HEPES/NaOH(pH 7.5), 20 mM sodium sulfate. The standard deviations were indicated.

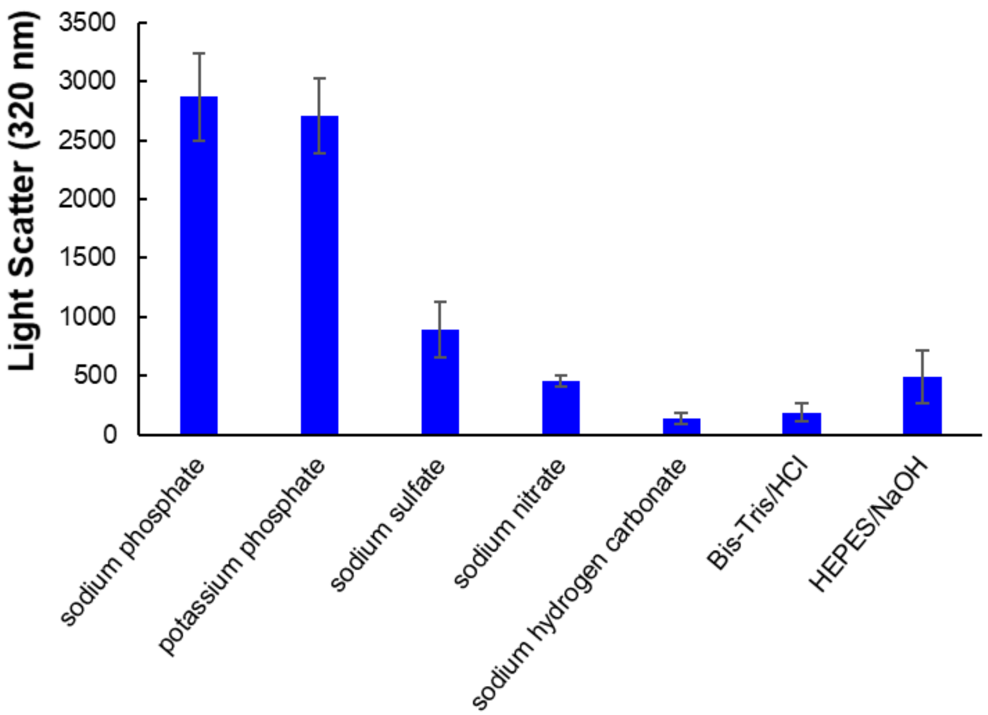

Supplementary Experimental Section for Figure S3

The final 400 μM of hSTOM(SPFH) was first dialyzed against the buffers indicated above, lyophilized, and then dissolved by deionized water. All samples were processed by sonication for 5 sec after gentle pipetting. Scattered light was measured at both excitation and measurement wavelengths 320 nm using a fluorophotometer F-7000 (Hitachi) with a 10 mm four-face polished quartz square cuvette. For all samples, measurements were performed three times.

Supplementary Figure S4

Overlay of HSQC spectra of hSTOM(SPFH) under different buffer conditions. Black: phosphate buffer solution, Red: Bis-Tris buffer solution. The peaks are shifted for some residues. Residues with $\Delta\delta$ >0.015 ppm were highlighted in orange.

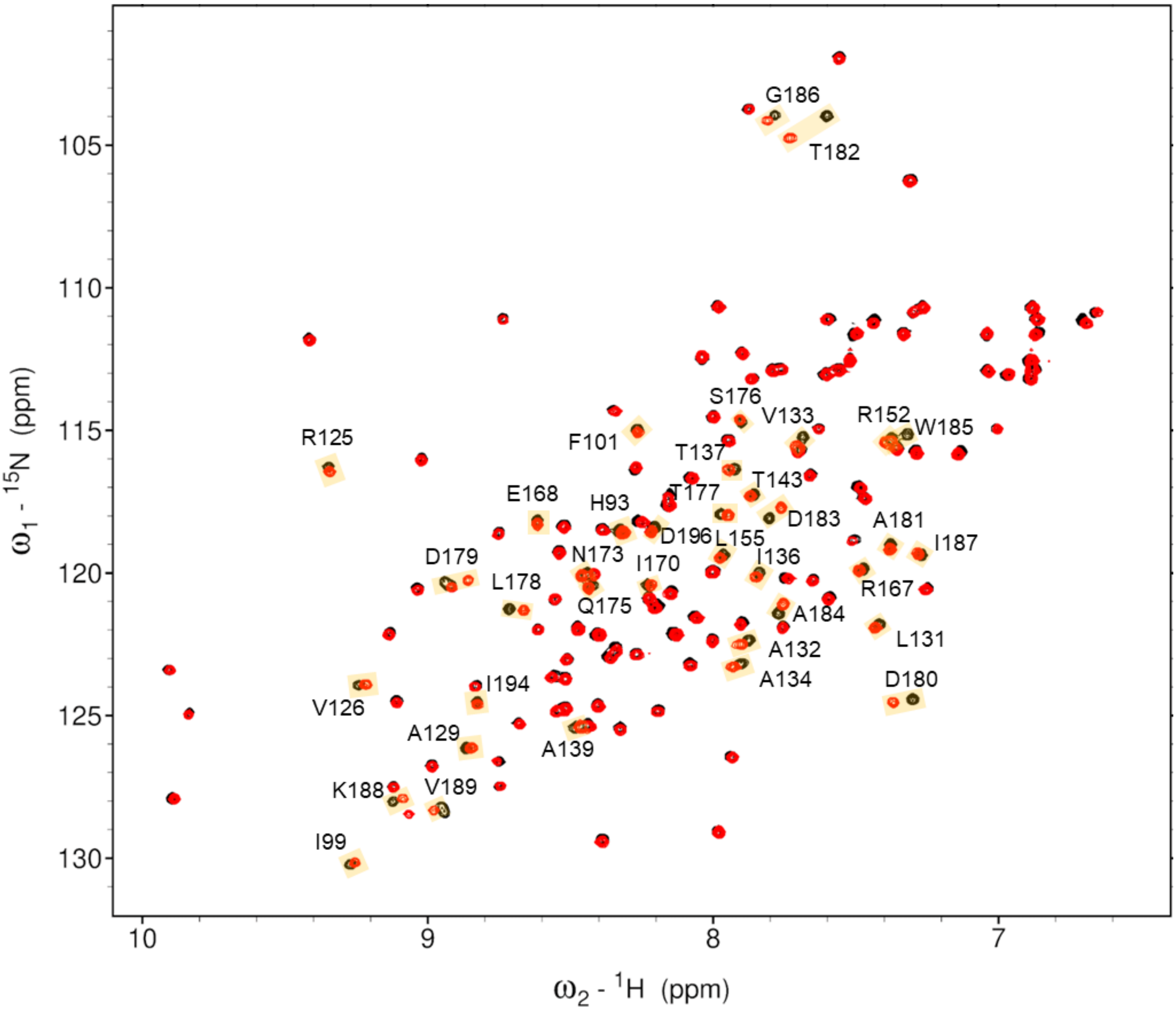

Supplementary Figure S5

Effect of phosphate ion upon formation of precipitates during centrifugal concentration of hSTOM(SPFH). The supernatants and precipitates were analyzed by 12.5% SDS-PAGE. M; marker, Lane 1, 3; samples dissolved in phosphate buffer, and Lane 2, 4; Bis-Tris buffer.

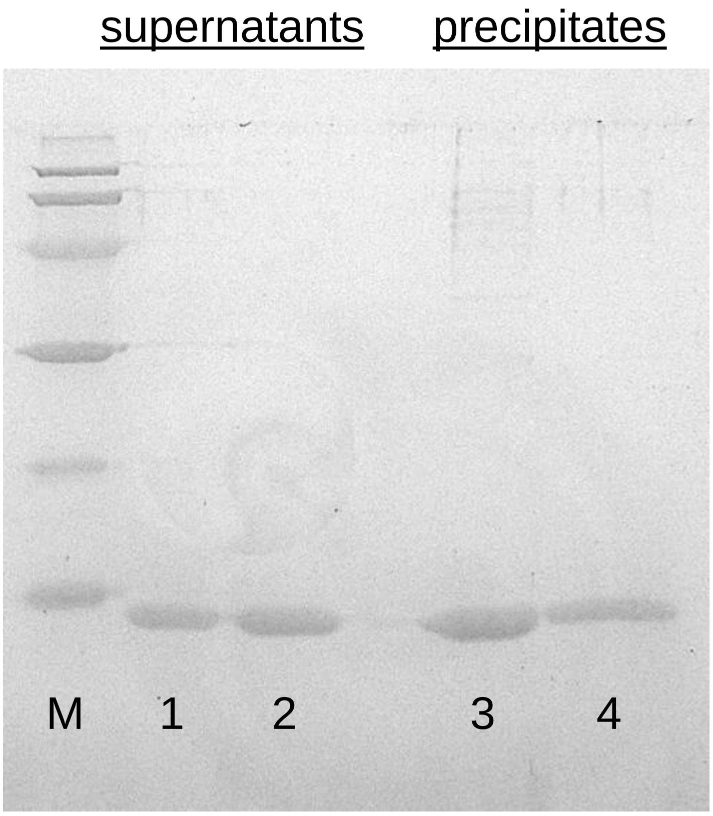

Supplementary Experimental Section for Figure S5

Approximately 50 µM of hSTOM(SPFH) was first dialyzed against the buffer containing 50 mM sodium phosphate (pH 7.0), 100 mM NaCl or 50 mM Bis-Tris/HCl (pH 7.0), 100 mM NaCl. The protein concentration of the sample was adjusted to 42 µM, then the aliquots were placed to the centrifugal ultrafiltration devices Microsep 3K (PALL, Port Washington, NY), that was arranged with a horizontal membrane. The membranes were rinsed with the same buffer before use. 4 mL of the protein samples were placed and centrifuged for 30 min x2. The samples above the membrane were carefully separated to supernatants and precipitates. The precipitates on the membranes were suspended to 200 µL of buffers and dissolved by sonication for 2 sec. The samples were analyzed by SDS-PAGE and stained with coomassie brilliant blue. The density of hSTOM(SPFH) bands were quantified by ImageJ and the results are as follows, lane 1; 6.8×10^5, lane 2; 8.5×10^5, lane 3; 10.3×10^5, and lane 4; 7.7×10^5.
